# A ^19^F magnetic resonance imaging-based diagnostic test for bile acid diarrhea

**DOI:** 10.1007/s10334-018-0713-9

**Published:** 2018-11-01

**Authors:** Jean-Pierre Raufman, Melissa Metry, Jessica Felton, Kunrong Cheng, Su Xu, James Polli

**Affiliations:** 1 Division of Gastroenterology and Hepatology, Department of Medicine, and Marlene and Stewart Greenebaum Comprehensive Cancer Center, University of Maryland School of Medicine, and the VA Maryland Healthcare System, Baltimore, MD 21201 USA; 2 Department of Pharmaceutical Sciences, University of Maryland School of Pharmacy, Baltimore, MD 21201 USA; 3 Department of Surgery, University of Maryland School of Medicine, Baltimore, MD 21201 USA; 4 Department of Diagnostic Radiology and Nuclear Medicine, University of Maryland School of Medicine, Baltimore, MD 21201 USA

**Keywords:** ^19^F MRI, Diarrhea, Irritable bowel syndrome, Bile acids, Gallbladder, Enterohepatic circulation

## Abstract

In up to 50% of people diagnosed with a common ailment, diarrhea-predominant irritable bowel syndrome, diarrhea results from excess spillage of bile acids into the colon—data emerging over the past decade identified deficient release of a gut hormone, fibroblast growth factor 19 (FGF19), and a consequent lack of feedback suppression of bile acid synthesis as the most common cause. ^75^Selenium homotaurocholic acid (SeHCAT) testing, considered the most sensitive and specific means of identifying individuals with bile acid diarrhea, is unavailable in many countries, including the United States. Other than SeHCAT, tests to diagnose bile acid diarrhea are cumbersome, non-specific, or insufficiently validated; clinicians commonly rely on a therapeutic trial of bile acid binders. Here, we review bile acid synthesis and transport, the pathogenesis of bile acid diarrhea, the reasons clinicians frequently overlook this disorder, including the limitations of currently available tests, and our efforts to develop a novel ^19^F magnetic resonance imaging (MRI)-based diagnostic approach. We created ^19^F-labeled bile acid analogues whose in vitro and in vivo transport mimics that of naturally occurring bile acids. Using dual ^1^H/^19^F MRI of the gallbladders of live mice fed ^19^F-labeled bile acid analogues, we were able to differentiate wild-type mice from strains deficient in intestinal expression of a key bile acid transporter, the apical sodium-dependent bile acid transporter (ASBT), or FGF15, the mouse homologue of FGF19. In addition to reviewing our development of ^19^F-labeled bile acid analogue-MRI to diagnose bile acid diarrhea, we discuss challenges to its clinical implementation. A major limitation is the paucity of clinical MRI facilities equipped with the appropriate coil and software needed to detect ^19^F signals.

## Introduction

Advances over the past two decades greatly expanded our understanding of how tight regulation of bile acid synthesis and transport maintains homeostasis of the bile acid pool. In concert with these advances, it became apparent that up to one-half of persons diagnosed with a common chronic gastrointestinal ailment, diarrhea-predominant irritable bowel syndrome (IBS-D), actually have bile acid diarrhea resulting from disruption of that homeostasis [1–3]. The exact prevalence of IBS-D in the United States (US) is uncertain, but estimates range from 7 to 16% of the population depending on which age cut-offs and criteria for diagnosing IBS-D are used [4]. Based on the current US population, these data suggest up to 20 million Americans may have bile acid diarrhea, a potentially life-long disorder that can profoundly diminish the quality of life, and impair daily activities and work performance, thus increasing both indirect and healthcare-related costs [5].

Despite the evident need, current approaches to diagnose bile acid diarrhea remain limited. To address this unmet clinical need, we conceived and developed ^19^F-labeled bile acid magnetic resonance imaging (MRI), a test that requires neither venipuncture nor exposure to ionizing radiation. In this review, we provide an overview of the mechanisms and regulation of bile acid synthesis and transport, and review the pathogenesis of bile acid diarrhea, current diagnostic options, and our efforts to develop and bring to the clinic a novel ^19^F magnetic resonance-based approach to diagnose bile acid diarrhea.

## Synthesis and enterohepatic circulation of bile acids

**Fig. 1:**
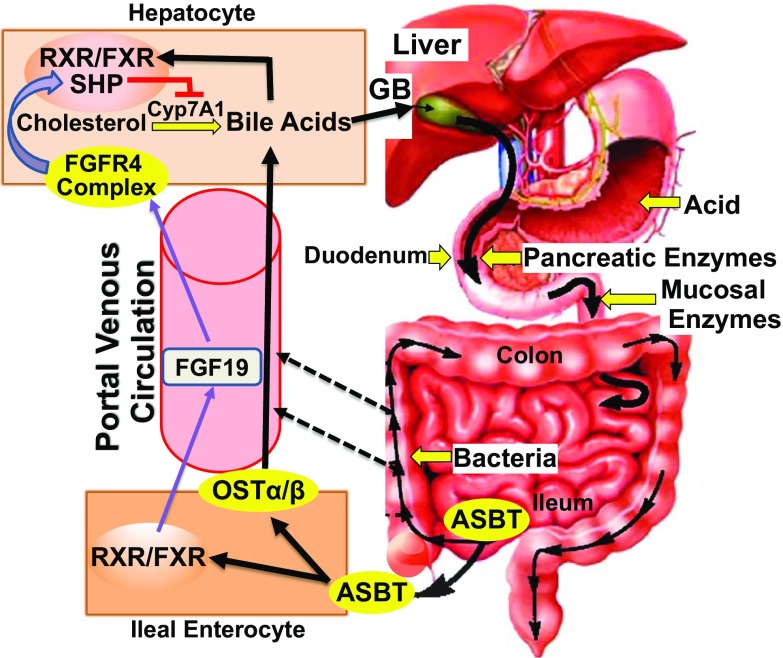
Enterohepatic circulation of bile acids. Black arrows trace the route of bile acids through the enterohepatic circulation, a highly efficient process that recovers > 95% of enteric bile acids for reprocessing (conjugation) and re-excretion by hepatocytes in the liver. Hepatocytes synthesize and excrete bile acids, byproducts of cholesterol metabolism, along with recycled, conjugated bile acids, into the bile ducts for storage in the gallbladder. Eating stimulates gallbladder contraction and bile acid release into the duodenum where they play a major role in fat digestion and absorption. In the ileum, ASBT actively transports bile acids into enterocytes where, via activation of nuclear FXR, bile acids stimulate FGF19 production and release into the portal venous circulation. The dashed arrows depict passive transport of bile acids from the intestines into the portal circulation. In hepatocytes, bile acid interaction with nuclear FXR and FGF19 interaction with the plasma membrane FGFR4 receptor complex repress Cyp7A1; feedback inhibition of bile acid synthesis. During transit through the gastrointestinal tract, native and modified bile acids are exposed to gastric acid, pancreatic and intestinal mucosal enzymes, and enteric bacteria.* ASBT* apical sodium-dependent bile acid transporter,* FGF19* fibroblast growth factor-19, *FGFR4* fibroblast growth factor receptor-4,* FXR* farnesoid X receptor,* GB* gallbladder,* OST* organic solute transporter,* RXR* retinoid X receptor,* SHP* small heterodimer partner

The enterohepatic circulation provides a highly efficient process for recycling both newly synthesized and conjugated bile acids; normally, less than 5% of bile acids released into the small intestine are lost in the feces (Fig. 1). In hepatocytes, cholesterol metabolism results in the synthesis of sufficient levels of bile acids to compensate for the amount excreted in the feces. Hepatic Cyp7A1 is the rate-limiting enzyme for the primary metabolic pathway of bile acid production. Newly synthesized and recycled bile acids, transported from hepatocytes into the bile ducts, are stored and concentrated in the gallbladder (Fig. 1). Eating stimulates gallbladder contraction and release of highly concentrated bile acids into the duodenum, where they achieve critical micellar concentrations and play a major role in the digestion and absorption of fats and fat-soluble vitamins.

In the ileum, the apical sodium-dependent bile acid transporter (ASBT) actively transports luminal bile acids into enterocytes (Fig. 1, lower left), where they bind and activate the nuclear farnesoid X receptor (FXR), which forms a complex with the retinoid X receptor (RXR). The activated FXR complex stimulates production and release into the portal venous circulation of fibroblast growth factor 19 (FGF19) (Fig. 1). FGF19, a gut hormone, circulates to the liver where it interacts with the fibroblast growth factor receptor-4 (FGFR4) complex on hepatocytes (Fig. 1, upper left). The activated FGFR4 complex then stimulates a nuclear small heterodimer partner (SHP) of hepatic FXR to repress Cyp7A1 expression—resulting in feedback inhibition of hepatic bile acid synthesis.

In addition, in ileal enterocytes (Fig. 1, lower left), organic solute transporters (OSTα and β) actively transport bile acids into the portal venous circulation for return to the liver. Similar to the actions of FGF19, binding of bile acids to hepatic FXR activates SHP to repress Cyp7A1. Thus, in hepatocytes, the combined actions of FGF19 and recycled bile acids provide redundant, possibly potentiating, mechanisms of feedback repression of bile acid production. This likely safeguards the liver against the damaging effects of toxic levels of bile acids that can occur in primary biliary cholangitis and other conditions resulting in cholestasis.

## Bile acid diarrhea and other potential adverse effects of increased levels of fecal bile acids

Increased levels of fecal bile acids may have several deleterious effects that may be both acute and chronic. In the colon, increased luminal levels of dihydroxy bile acids may induce diarrhea by several mechanisms, including stimulation of electrolyte (particularly sodium), water and mucus secretion, and damage to the mucosal surface. Collectively, these adverse effects increase mucosal permeability and stimulate intestinal motility and the frequency of defecation.

Abundant epidemiological findings collected over many years and the results of animal studies strongly support the conclusion that chronic exposure of the colon epithelium to increased levels of fecal bile acids promotes dysplasia and neoplasia [6, 7]. Using two animal models described later in this review, the authors employed a commonly used model of colon neoplasia to show that even modest increases in fecal bile acid concentrations over an extended period (4–5 months) can augment colon neoplasia [8, 9]. Thus, diagnosing and treating bile acid diarrhea is likely to provide both short- and long-term clinical benefits.

Conditions that diminish ileal uptake of intestinal bile acids may increase their spillage into the colon. Examples include inflammatory diseases (e.g., Crohn’s disease) or surgery involving the ileum; the latter may be a consequence of ischemia or trauma. Certain classes of US Food and Drug Administration (FDA)-approved drugs also have the potential of impairing ASBT transport function [10]. Thereby, these drugs may also increase bile acid spillage into the colon.

Surprisingly, defective function of ASBT as a consequence of mutation or lack of expression is uncommon [11]. A recent report suggests that OST mutation can also cause congenital chronic diarrhea, but this also appears to be rare [12]. Instead, the foremost cause of excess spillage of bile acids into the colon and resulting bile acid diarrhea appears to be defective regulation of bile acid production and gallbladder filling by a deficit of ileal FGF19 [2]. In the absence of feedback repression of hepatic Cyp7A1 activity by the FGF19/FGFR4 axis, increased hepatic production of bile acids and decreased filling of the gallbladder result in excess bile acid levels in the ileum that overwhelm ASBT transport capacity [2, 13, 14]. This mechanism is similar to that underlying the transient diarrhea commonly observed following cholecystectomy; removal of the gallbladder commonly results in the unregulated spillage of bile acids into the small intestine.

Without a means to replace FGF19 or otherwise mimic this mechanism to repress bile acid production, clinicians commonly treat bile acid diarrhea using chemical sequestrants to bind bile acids, an approach that can be complicated by drug-induced bloating, abdominal discomfort, flatulence, and constipation. If patients take bile acid binders at the same time as other drugs, these agents can sequester medications and diminish their bioavailability and therapeutic efficacy. Thus, it is important to establish the correct diagnosis before committing patients of potentially life-long treatment with bile acid sequestrants.

## Current approaches to diagnosing bile acid diarrhea

In Table 1, we summarized the key features of current approaches to diagnose bile acid diarrhea. In parts of Europe, the United Kingdom and Canada, the most common approach is to use radionuclide imaging to measure retention of an orally administered radiolabeled bile acid, ^75^Selenium homotaurocholic acid (SeHCAT) [15]. Despite being the ‘gold standard’ in these countries for more than 25 years, for reasons that are not entirely clear, SeHCAT testing is not FDA-approved for use in the US; this is not likely to change.

**Table 1:** Current tests to diagnose bile acid diarrhea

Test	Brief description of methods	Disadvantages	Sensitivity/specificity; references [18, 19, 33]
^72^SeHCAT retention	Methods and interpretation vary. Commonly, subjects ingest ^72^SeHCAT and undergo a gamma camera body scan immediately and again 7 days later. At 7 days, normal subjects retain ≥ 15% of the initial ^72^SeHCAT signal	Not available in many countries (not FDA-approved in the US). Rapid intestinal transit may result in false positives	89%/100%; considered ‘gold standard’ when available
Fecal bile acid levels	Methods and interpretation vary. Subjects collect stool for 48–72 h while ingesting at least 100 g/day dietary fat. Fecal bile acid levels > 2 mmol/24 h are considered elevated	Cumbersome. Technically difficult. Not commercially available	Unknown
Serum FGF19	Blood draw after overnight fast	Requires research lab: not commercially available. Insufficiently validated	58%/84%
Plasma C4	Blood draw after overnight fast	Requires research lab. Results altered in liver disease or with some medicines. Insufficiently validated	90%/79%
Trial of bile acid binder	Gage therapeutic response to 2- to 4-week treatment with colestipol, cholestyramine, or colesevalam	Interference with drug absorption. Bloating, flatulence, abdominal pain, and constipation	Unknown

Until recently, guidelines for the management of IBS-D published by major US clinical societies commonly neglected to acknowledge the possibility of bile acid diarrhea [16]—this appears to be changing [17, 18]. In addition to delays in distributing information about the potential role of bile acids, some have attributed lack of access to SeHCAT testing [2] as another reason for the common failure of US physicians to consider excess spillage of bile acids into the colon as a cause of chronic diarrhea.

Directly measuring fecal bile acids requires a restricted diet, a cumbersome 3-day stool collection, and an experienced research laboratory with normative data; this test is unavailable in most hospitals and clinics. Measuring plasma levels of C7α-hydroxy-4-cholesten-3-one (C4), which correlate with hepatic bile acid production, is less sensitive and specific than SeHCAT testing, can be confounded by diurnal variation and clinical factors, and is also not widely available [18]. Measuring serum levels of FGF19 is possible but faces similar limitations to that for C4 testing and requires additional validation before clinical use. Although investigators reported statistically significant inverse correlations between C4 and FGF19 levels, these tests were not evaluated with respect to fecal bile acid levels and clinical outcomes [19, 20].

In the US, although the primary diagnostic approach to bile acid diarrhea remains a non-standardized therapeutic trial of a bile acid binder, the optimal sequestrant (e.g., cholestyramine, colestipol, or colesevelam), dose, and duration of the therapeutic trial remain undefined. Neither the sensitivity nor specificity of this test has been determined; diarrhea from many causes may respond non-specifically to bile acid binder treatment. A therapeutic trial also risks the side effects of bile acid binder treatment described above. In general, their difficulty of use, lack of ready availability, excessive cost, side effects, and want of validation in a cohort sufficiently enriched in subjects with bile acid diarrhea limits each of these available tests. The lack of availability of SeHCAT and limitations of other tests, as well as advances in fluorine-based magnetic resonance imaging, led us to conceive of using ^19^F-based magnetic resonance imaging to visualize more precisely the transport of ^19^F-labeled bile acids.

## Development of ^19^F-labeled bile acid analogues for MRI

**Fig. 2:**
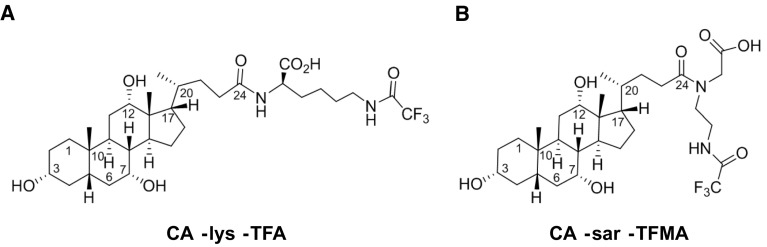
Chemical structures of ^19^F-labeled bile acid analogues. **a** CA-lys-TFA, a trifluoroacetyl l-lysine conjugate of cholic acid, forms a secondary amine at the C-24 region with the amino acid lysine that links the three equivalent ^19^F atoms to cholic acid. **b** CA-sar-TFMA, a trifluoro-*N*-methyl-acetamide conjugate of cholic acid, forms a more stable tertiary amine with the amino acid sarcosine at the C-24 region that links the three equivalent ^19^F atoms to cholic acid

We designed and evaluated ^19^F-labeled bile acids for their ability to mimic endogenous bile acids and their transport, concentrate in the gallbladder, and produce a quantifiable concentration-dependent ^19^F-MRI signal. We developed bile acid analogues consisting of the same basic structure—we conjugated three equivalent fluorine (^19^F) atoms to the native human primary bile acid, cholic acid, via an amino acid linker. We chose cholic acid, a highly potent substrate of ASBT, since it has a relatively high aqueous solubility and low plasma protein binding compared to other bile acids. This is favorable for ^19^F-MRI, since for detection the bile acid analogues have to be in solution and unbound. Like native bile acids, these bile acid analogues were designed to carry a negative charge in the C-24 region, via the carboxylate, which enhances bile acid interaction with ASBT [21]. Conjugation of bile acids at the C-24 region with an amino acid, whether glycine or taurine, generally enhances their affinity for and transport capacity by ASBT [22]. Based on these structural considerations, we synthesized and evaluated trifluoroacetyl l-lysine and trifluoro-N-methyl-acetamide conjugates of cholic acid, referred to as CA-lys-TFA and CA-sar-TFMA, respectively (Fig. 2).

Amongst the many hurdles to drug design, to reach ASBT, the target transporter in the distal ileum, ^19^F-labeled bile acid analogues have to withstand the harsh gastrointestinal environment, including gastric acid, pancreatic and small intestinal mucosal enzymes, and gut flora (Fig. 1, right). We designed CA-sar-TFMA as an improved bile acid analogue compared to CA-lys-TFA, which is not resistant to bacterial deconjugation by cholylglycine hydrolase, an enzyme primarily found in the colon and terminal ileum [23]. The formation of a tertiary amine via the C-24 carboxylate, which attaches cholic acid to the amino acid sarcosine (Fig. 2b), provided a more stable bond in vitro compared to that of the secondary amine in the structure of CA-lys-TFA, which attaches the bile acid to lysine (Fig. 2a) [23, 24]. Surprisingly, despite enhanced in vitro stability of CA-sar-TFMA over CA-lys-TFA [23], this was not apparent in vivo in mice, where the two showed similar stability and distribution [25]. The basis for these in vitro and in vivo differences remains unknown.

Additionally, the structural properties of CA-sar-TFMA and CA-lys-TFA permit their transport within the enterohepatic circulation since they are substrates for both ileal ASBT and the hepatic Na+/taurocholate co-transporting polypeptide (NTCP). Despite their different structures, these bile acid analogues performed alike, sufficiently to assess impaired bile acid transport within the enterohepatic circulation [25]. In our studies, incubation of CA-sar-TFMA and CA-lys-TFA with stool and gallbladder homogenates for up to 24 h revealed no significant degradation in either ^19^F-bile acid analogue [25].

CA-sar-TFMA and CA-lys-TFA concentrated in the gallbladder above the ^19^F-MRI limit of detection of 2.27 mM of bile acid analogue, as there are three equivalent fluorine atoms in each structure. The ^19^F-MRI limit of detection was determined to be the noise magnitude plus 2.5 times the noise standard deviation, calculated from a region of interest (ROI) near the periphery of the image [25]. Using this method, there is greater than 99% confidence that voxels containing bile acid analogue concentrations above 6.82 mM relative to the phantom are from actual ^19^F signal, and not noise. This limit of detection was similar to that estimated for isoflurane [26]. We used liquid chromatography–mass spectrometry (LC–MS) to measure isoflurane in the gallbladder. LC–MS has an exceptionally low limit of detection (i.e., many of orders of magnitude less than 1 mM). LC–MS quantifications were compared to the ability of ^19^F MRI imaging to detect isoflurane. Although ^19^F-MRI quantification, including limit of detection, is a function of many variables, there was general agreement that ^19^F-MRI limit of detection was on the order of a few millimolar of tri-fluorinated probe compound. It is important to recognize that the MRI limit of detection is not static. It may vary as a function of many factors including field strength, imaging sequence/parameters, relaxation times, and others.

## In vivo testing of ^19^F-labeled bile acid analogue-MRI in mice with defective intestinal transport of bile acids

Between meals, bile acids accumulate and are concentrated in the gallbladder. Compared to other organs comprising the bile acid pool, bile acid concentrations are orders of magnitude greater in the gallbladder (millimolar compared to micromolar levels in other compartments). Thus, we reasoned the gallbladder would be the most promising organ in which to visualize ^19^F-labeled bile acid analogues by MRI. We also reasoned we could validate MRI measurements by applying analytical techniques to measure ^19^F-labeled bile acid levels directly in gallbladder contents.

**Fig. 3:**
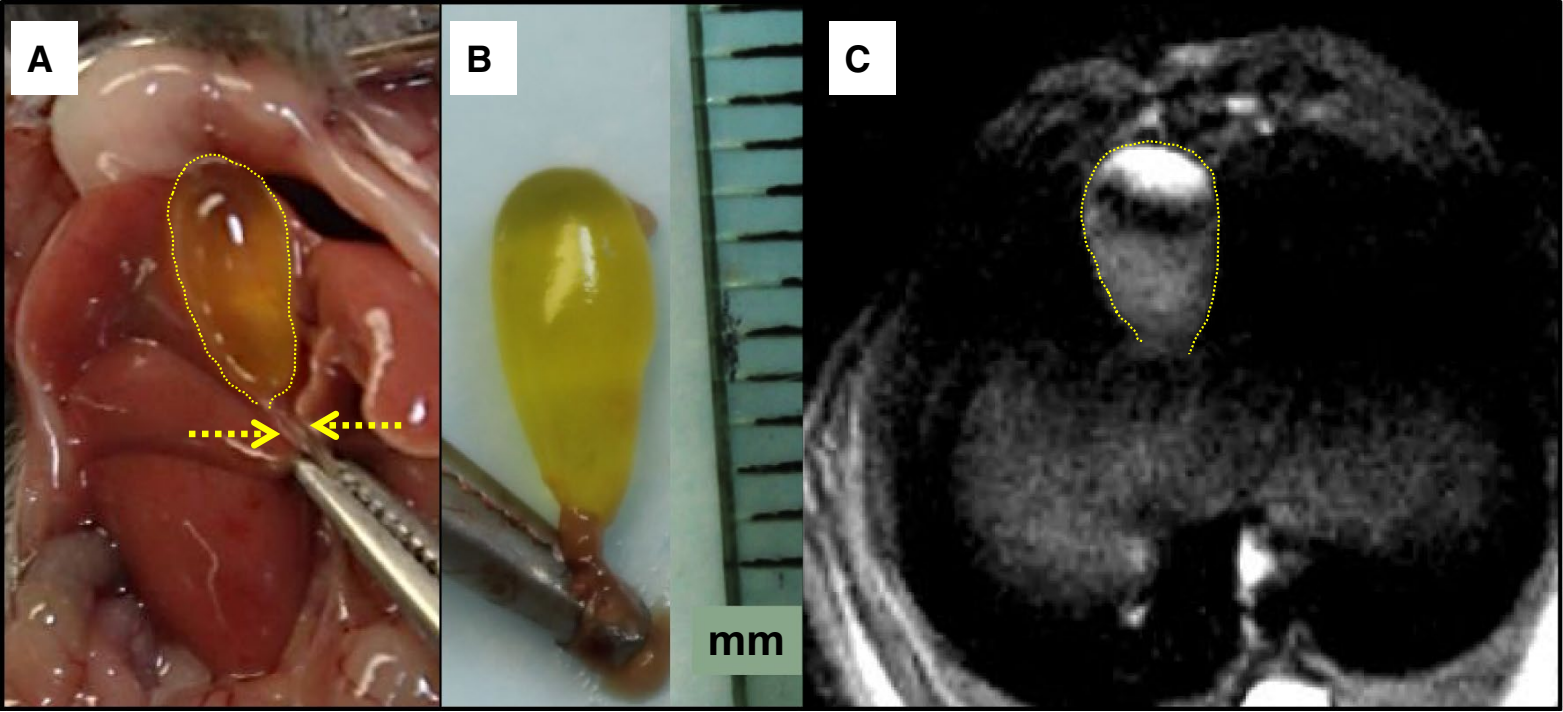
In vivo and ex vivo anatomical and in vivo ^1^H MRI images of the mouse gallbladder. **a** Exposed mouse gallbladder after abdominal incision. The yellow dotted line indicates the bile-filled fasting gallbladder; the dashed arrows indicate the clamped common bile duct. **b** Excised intact gallbladder with the common bile duct clamped. The ruler is marked in millimeters (mm). **c** High-resolution proton density-weighted MRI image of the fasting murine gallbladder (yellow dotted line)

**Fig. 4:**
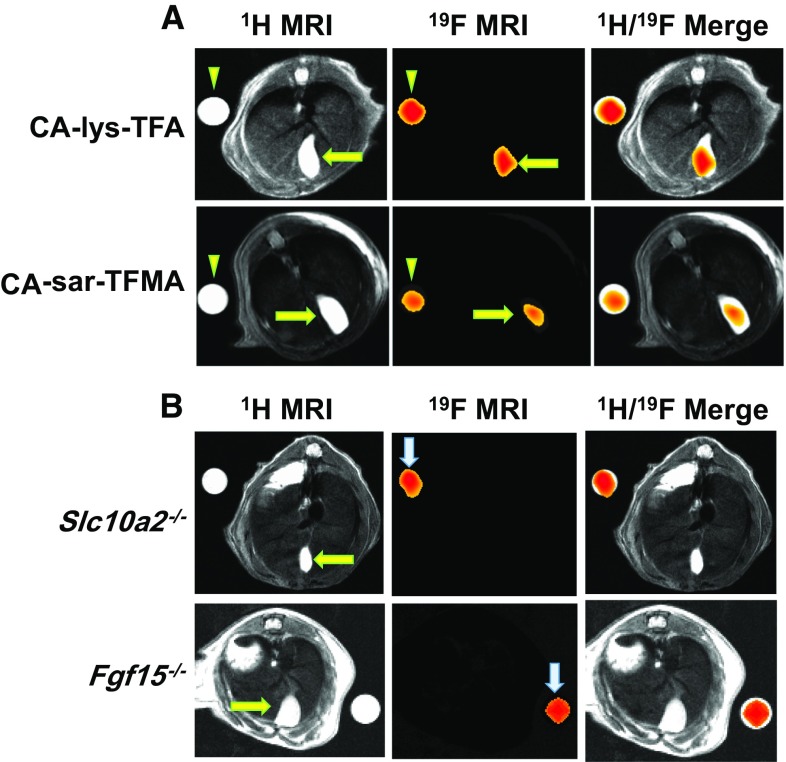
Magnetic resonance imaging of ^19^F-labled bile acid analogs in wild-type mice, and mice with knockout of a key bile acid transporter, ASBT, or of FGF15, the mouse homologue of FGF19. **a** Representative MR images from mice gavaged with either 150 mg/kg CA-lys-TFA (top) or CA-sar-TFMA (bottom). We used CA-lys-TFA- and CA-sar-TFMA-containing phantoms (arrowheads) adjacent to the mice to extrapolate the compound concentrations within the gallbladders (24 mM CA-lys-TFA; 34.2 mM CA-sar-TFMA). The left panels show ^1^H MR images of mouse abdominal cross-sectional anatomy with the gallbladders indicated by arrows. Middle panels show ^19^F MR images obtained from the same cross-sectional area. Right panels show merged images ^1^H and ^19^F MR images. **b** Representative MR images from mice deficient in ASBT (*Slc10a2*^−*/*−^; top) and FGF15 (*Fgf15*^−*/*−^; bottom) after gavage with 150 mg/kg CA-lys-TFA. Whereas the CA-lys-TFA phantoms provide ^19^F signals (arrows), ^19^F signals are not detected emanating from the gallbladders of either knockout mouse

To achieve these goals, we developed surgical methods for clamping the common bile duct and excising mouse gallbladders with their contents intact, illustrated in a video we created for the *Journal of Visualized Experiments* [27] (Fig. 3). We purchased surface and linear polarized volume ^1^H/^19^F radio frequency (RF) coils and the software needed (Bruker BioSpin MRI GmbH, Germany) to achieve dual ^1^H/^19^F MRI of the mouse gallbladder (Figs. 3, 4); detailed information on the hardware is provided in Ref. [26]. For proof-of-concept experiments, we sought fluorinated drugs that undergo first-pass metabolism in the liver and excretion, either in native or modified form, into the bile for storage and concentration in the gallbladder. Fortuitously, we discovered that isoflurane, a tri-fluorinated inhalation anesthetic commonly used to anesthetize mice for experimental procedures, was such a drug. In pilot experiments, we demonstrated MRI imaging could detect the accumulation of inhaled isoflurane in the gallbladders of live mice and validated these measurements by excising gallbladders and assaying contents by liquid chromatography–mass spectrometry/mass spectrometry (LC–MS/MS) [26].

Next, we tested the ability of ^19^F MRI to detect gallbladder accumulation of the two ^19^F-labeled bile acid analogues, CA-lys-TFA and CA-sar-TFMA. After gavage with either analogue, we detected robust ^19^F signals emanating from the gallbladders of WT mice (Fig. 4a) [23, 28, 29].

To demonstrate the potential utility of this ^19^F magnetic resonance-based approach to diagnose bile acid diarrhea, we employed mouse strains with deficient intestinal expression of the key bile acid transporter, ASBT, or of FGF15, the mouse homologue of FGF19; investigators commonly use FGF15-deficient mice to mimic human FGF19 deficiency [14, 30]. As shown by the summary of published data shown in Table 2, fecal bile acid levels are elevated six- to ten-fold and two- to three-fold in ASBT- and FGF15-deficient mice, respectively [30–32].

**Table 2:** Increased fecal bile acid levels in ASBT- and FGF15-deficient mice

Mouse model	Fecal bile acids			References
	WT mice (µmol/day/100 g bw)	Knockout mice (µmol/day/100 g bw)	Fold-increase (KO/WT)	
Asbt^−*/*−^	Males: ~ 6^a^	~ 138^a^	24	[31]
	Females: ~ 9^a^	~ 98^a^	11	
	23.3 ± 2.1	110.0 ± 10.6	4.7	[32]
Fgf15^−/−^	~ 17.5^a^	~ 35.0^a^	~ 2	[30]
	15.24 ± 1.00	34.45 ± 3.52	2.26 ± 0.28	[34]

*WT* wild-type, *bw* body weight, *KO* knockout

^a^Numbers derived from bar graphs in the referenced publications

Compared to WT mice, we anticipated that after gavage, both ASBT- and FGF15-deficient mice would have reduced ^19^F-labeled bile acid analogue levels in the gallbladder. In ASBT-deficient mice, this would be a consequence of diminished ileal uptake of all bile acids, including ^19^F-labeled bile acid analogues. In FGF15-deficient mice, we reasoned the expanded intestinal compartment of the bile acid pool resulting from unbridled bile acid synthesis would dilute ^19^F-labeled bile acid analogues in the gut and there would be increased competition for uptake by ASBT; these effects would impede ^19^F-labeled bile acid analogue uptake into the enterohepatic circulation.

As shown in Fig. 4b, after gavaging mice with the same MRI settings and dose of CA-lys-TFA used for imaging WT mice (Fig. 4a), investigators masked to mouse genotype were unable to detect ^19^F MRI signals emanating from the gallbladders of ASBT- and FGF15-deficient mice [25, 28]. Direct measurement of ^19^F-labeled bile acid analogues in the contents of gallbladders harvested from WT, and ASBT- and FGF15-deficient mice confirmed reduced levels in gallbladders from knockout mice [25, 28]. These proof-of-concept experiments reveal the potential of using ^19^F-labeled bile acid analogue—MRI to discriminate normal from defective bile acid transport (e.g., lack of ASBT) or feedback suppression of bile acid synthesis (e.g., lack of FGF15/19).

## Potential advantages and limitations of ^19^F-labeled bile acid analogue: MRI

^19^F-labeled bile acid analogue: MRI has several advantages compared to currently available tests for diagnosing bile acid diarrhea. These include the lack of ionizing radiation from either ^19^F-labeled bile acid analogues or MRI, and no need for venipuncture; one would administer ^19^F-labeled bile acid analogues orally and the test would not require blood collection. Although we have yet to perform formal toxicology testing, the initial experience with this method in mice supports the safety of single dosing at 150 mg/kg body weight [29].

Nonetheless, ^19^F-labeled bile acid analogue: MRI has potential limitations. The sensitivity and specificity of this test in humans or in a ‘real world’ situation are unknown. It is conceivable we could enhance the sensitivity of the test by adding additional NMR-equivalent fluorine atoms to the bile acid analogues, but we would have to ensure this modification did not impede the transport of the bulkier molecule and that it still mimicked that of native bile acids. As currently designed, ^19^F-labeled bile acid analogue—MRI would not work in persons with a non-functioning gallbladder or those who have undergone cholecystectomy. Addressing these limitations requires re-configuring the test, perhaps by determining how much ^19^F-labeled bile acid analogue remains in the body after a defined period, as is done in SeHCAT testing (Table 1).

## Challenges facing clinical implementation of ^19^F-labeled bile acid: MRI to diagnose bile acid diarrhea

Despite the promise that rapid diagnostic testing might considerably reduce the lag time between the start of symptoms and the correct diagnosis of bile acid diarrhea, thereby resulting in earlier treatment, implementation of new tests faces a slow regulatory approval process, uncertainties regarding insurance coverage, and initial resistance to new diagnostic approaches. Moreover, since the proposed fluorinated bile acid analogues are ‘drugs’, they require preliminary toxicity testing in animals and additional regulatory compliance to obtain an investigational new drug application (IND) from the US Food and Drug Administration.

After obtaining an IND, initial testing must address scaling the dose for larger organisms and determining the appropriate time-course for optimal visualization of a fluorine signal emanating from the gallbladders of normal volunteers. Using patients with known bile acid diarrhea, investigators must then determine the analytic validity of ^19^F-bile acid-MRI by its sensitivity (probability that someone with bile acid diarrhea will have no fluorine signal from the gallbladder) and specificity (probability of fluorine signals emanating from the gallbladders of healthy persons). Converse experiments will establish the positive and negative predictive values of the new test (probability that absence of a gallbladder signal indicates disease and probability that a gallbladder signal indicates absence of bile acid diarrhea, respectively). Optimally, one would compare these parameters to the results of other tests for bile acid diarrhea, e.g., SeHCAT or serum levels of FGF19 and C4. Admittedly, the unavailability of SeHCAT testing appears to pose an impregnable barrier to performing such head-to-head comparison in the US.

A specific challenge facing implementation of ^19^F-labeled bile acid analogue magnetic resonance imaging is the paucity of clinical MRI machines with the necessary hardware (multi-nuclear setting and ^1^H/^19^F RF coil) and software to detect ^19^F-labeled bile acid analogues. This general problem facing implementation of all ^19^F-based MRI will most likely require a financial incentive for radiology facilities to make the necessary investments.

## Conclusions

^75^Selenium homotaurocholic acid (SeHCAT) testing, considered the most sensitive and specific means of identifying individuals with bile acid diarrhea, is unavailable in many countries, including the US, and the remaining tests to diagnose bile acid diarrhea are cumbersome, non-specific, or insufficiently validated. Clinicians commonly rely on a therapeutic trial of bile acid binders, which has its own limitations.

To address this clinical need, we developed a novel ^19^F magnetic resonance imaging (MRI)-based approach to diagnosing bile acid diarrhea using newly created ^19^F-labeled bile acid analogues, CA-lys-TFA and CA-sar-TFMA, with in vitro and in vivo transport characteristics that mimic those of naturally occurring bile acids. Using dual ^1^H/^19^F MRI of the gallbladders of live animals fed CA-lys-TFA confirmed by LC–MS/MS analysis of gallbladder contents, we were able to differentiate WT from knockout mice deficient in intestinal expression of a key bile acid transporter, ASBT, or FGF15, the mouse homologue of FGF19. These findings suggest ^19^F-labeled bile acid analogue—MRI has potential as a novel diagnostic test for bile acid diarrhea. Nonetheless, its development for clinical use faces considerable challenges.
